# The mentored experience to enhance opportunities in research (METEOR) program

**DOI:** 10.1080/10872981.2021.2014290

**Published:** 2021-12-08

**Authors:** Lisa Schwartz, Naomi Luban, Alison Hall, Diane McQuail, Yolanda Haywood

**Affiliations:** a The George Washington University School of Medicine and Health Sciences, Ashburn, VA, USA; b Children’s National Hospital

**Keywords:** Medical students, mentoring, research, qualitative, URM

## Abstract

**Problem:**

Medical students from groups that are underrepresented in medicine are less likely to pursue careers that incorporate research as compared to their white peers. Clinical and Translational Science Award (CTSA)-funded institutions encouraged centers to establish short-term, mentored summer research opportunities to motivate students underrepresented in medicine to enroll in medical school and ideally choose a career that incorporates research into their clinical practice.

**Approach:**

The Mentored Experience To Enhance Opportunities in Research (METEOR) Program was established in 2012 in partnership with the Clinical and Translational Science Institute at Children’s National (CTSI-CN) and The George Washington University (GW) School of Medicine and Health Sciences. Rather than a single summer experience, the METEOR Program is innovative in that it is intended to support the success of participants throughout the duration of their medical school training and beyond.

**Outcomes:**

Scholarly output of participants of the first four cohorts included 23 empirical research articles in peer-reviewed journals, five review articles, eight case reports, one empirical research article in a student-led journal, one commentary in a professional journal, 20 university-based poster presentations, three national poster presentations, and one international poster presentation. Interviews revealed themes aligned with constructs of the Social Cognitive Career Theory. Overall mentorship was seen as a key component of the METEOR Program. In addition, the ability to come to campus prior to the start of medical school, as part of a cohesive cohort, along with the addition of lectures and field trips, further enhanced participants’ experiences.

**Next Steps:**

Our findings will be incorporated into improvements to the program for future cohorts and may inform the design of similar mentored research programs. With increased enrollment, quantitative studies of the effectiveness of the program are planned.

## Problem

Medical students who are underrepresented minorities (URM) are less likely to pursue careers that incorporate research as compared to their white peers [1]. Clinical and Translational Science Award (CTSA)-funded institutions are encouraged to establish short-term, mentored summer research opportunities to motivate URM to enroll in medical school and ideally choose a career that incorporates research into their clinical practice [2].

## Approach

The Mentored Experience To Enhance Opportunities in Research (METEOR) Program is innovative as it is intended to support the success of participants throughout the duration of their medical school training and beyond rather than a single summer experience. Students admitted to the MD program and identified as being URM as defined by the NIH criteria [3] are encouraged to apply. Each year METEOR Program directors, with the dean of MD admissions, select 2–5 METEOR students based on elements of their AMCAS application and an additional personal statement.

Based on past research experience and interest, each new METEOR student is matched with a mentor, who is a full-time faculty member and researcher but not necessarily URM. METEOR students work with their mentor during the summer preceding medical school, the summer between the first and second years of medical school, and during up to 12 weeks of a research elective in their fourth year of medical school. As opportunities arise and time permits, students are encouraged to work with their mentor during all four academic years.

METEOR students enroll in the clinical and translational research (CTR) scholarly concentration of the MD Program curriculum, which includes a monthly lecture series as well as a required research project. Students arrive in early summer prior to matriculation to the medical school campus and are provided university housing and a stipend. All METEOR students attend a weekly research lecture series during the pre-matriculation summer and participate in field trips to local institutions, including the National Institutes of Health, the National Library of Medicine, and the Food and Drug Administration. Informal social activities allow for networking among members of multiple cohorts and mentors.

## Outcomes

The impact of the program among the first four cohorts (N = 12) who entered the program in summers 2012 through 2015 was explored through identification of scholarly output in public databases and audiotaped semi-structured interviews. Students’ scholarly output was defined as peer-reviewed journal articles and posters and presentation citations at both university-based and national conferences. Databases (ORCiD, Scopus, PubMed, ResearchGate, Health Sciences Research Commons), search engines (Google Scholar, Google), and keyword searches were utilized to retrieve results.

Thematic analysis using NVivo 20.4.0 was performed on transcripts of the interviews of five participants using the Social Cognitive Career Theory (SCCT), a framework to examine mentoring relationships [4]. SCCT predicts that one’s interest in an activity will be enhanced when they see themselves as competent in such activities (*self-efficacy*), and the activity results in positive, valued outcomes (*outcomes expectation*).

Scholarly output from 2012 through 2020 for 10 out of the 12 participants was identified and included 23 empirical research articles in peer-reviewed journals, five review articles, eight case reports, one empirical research article in a student-led journal, one commentary in a professional journal, 20 university-based poster presentations, three national poster presentations, and one international poster presentation. Notably numerous university-based poster presentations ultimately led to peer-reviewed publications.

Qualitative analysis of interview transcripts revealed numerous themes (Table 1). Mentors were critical in the students’ increased self-efficacy in the research process:

It certainly helped when I went to both publish or present, because when I worked with my mentor, we went through my entire presentation slide by slide, and we talked about, “Okay, this is what is important; this is what you need to talk about; this is a target audience; you have to make sure you incorporate this; this is what they need to know.” … So that was really helpful moving forward, because then I was able to replicate that particular guide, and it helped with a later presentation. (Cohort 1)

**Table 1.** Representative quotes from qualitative interviews aligned with themes

Theme/ Construct	
Self-Efficacy	I don’t think that if I hadn’t had the experience with our METEOR mentors that I would have been able to kind of navigate through how to even start a project, just knowing the basics of working in a lab, being able to orient yourself, ask for help when you need it. I think that that aspect of the METEOR program was very helpful. (Cohort 1) The summer between my first and second year I actually spent a lot more time actually doing data collection for the digital research project in pediatric oncology, and understanding how to use online resources like REDCap and what it really meant to dive into, what it takes to not just be part of the research study, but how data-collection works and all of that as well. (Cohort 3) I did publish while I was in [previous] research, but [METEOR] definitely helped me develop my researching skills. I did publish in medical school and I’m definitely seeing that the skills that I learned in the program, in terms of IRB meetings that I sat in on and watching that process of how to apply for IRBs, how to edit papers, and how to conduct a research study, really helped me in terms of the future research that I did [in residency]. (Cohort 3) I still learned about writing a manuscript from start to finish and submitting abstracts and working with a multi-disciplinary team, so working with pharmacists and statisticians and specialists in other fields, to get a project from start to finish. So I think I definitely took away many skills from these projects working with my mentor that was assigned to me through the METEOR program. (Cohort 4) I think I learned a lot through the program. This is my first time that I was able to write a manuscript from start to finish as the first author that ended up being accepted and published, so I learned all about that process. I had done poster presentations in the past but never at national conferences, so I think I also learning that skill of being able to explain your research to people that are specialized in that area, versus just generalized people, that actually are going to be asking thoughtful and relevant questions because this is their field and this is what they’re interested in, and so I think I definitely gained a lot of valuable experience in that sense. (Cohort 1) He taught me so much in terms of getting a manuscript published, I think a lot to learn there in terms of the editing and the sending back to the journal and then getting revisions back and doing that. There was a lot of learning to be had there in terms of publishing an actual paper. (Cohort 2)
Outcome Expectations	I think METEOR helped in terms of giving me the exposures to all the different types of research and knowing that there’s more than just bench research out there, and clinical translational research that would affect the patient, and that would be something that I’d be most interested in pursuing. (Cohort 2) So I think that the METEOR program significantly impacted my decision now to continue with research, just because I was able to see firsthand how a physician that does … that is engaged. is strongly tied to research, is able to go about their day, their day-to-day, conducting their research but then also practicing medicine. (Cohort 1)
Broader Exposure to the Research Process	The ins and outs of basically trying to establish how I’m going to not only conduct my research but how I will go about getting funding if I needed it, and I think that was helpful. (Cohort 1) In terms of how you actually recruit patients for a study, and there was another group in the hospital that was competing for kind of a similar cohort of patients, and so how that goes, and then on top of that the collaboration; I had a lot of partners and just people in the lab and how to work with the lab and the protocols and actually doing the stuff hands-on. (Cohort 2) I hadn’t done that before, like trying to recruit other people to help me on my study; I hadn’t done that before. That was all new. It was less of basic research skills and more so just the business of getting research done, and the perseverance to try to push to get things done was a new experience for me. (Cohort 1) Working with this particular mentor I was able to see how research directly impacted medicine, so what she was working with technology, I could see it cross over into her realm of her clinical work … It was nice to see the correlation between the bench work and then it being applied directly to the clinical aspect of the research. (Cohort 1)
Expanded Networks	Some research meetings within the department that had nothing to do with [my mentor]. They were just people that she introduced me to, and they had some research, and they invited me to come to their meeting. (Cohort 2) [My mentor] introduced me to people that helped me develop my researching skill even more so. (Cohort 3) [My mentor’s] been so helpful in terms of getting through med school and getting me ready for residency, but then also is able to help connect me to many people in the field that I’m now in. (Cohort 4) I think [my mentor] definitely helped me out in that he was able to put me in contact with the important people in those areas and just help me along and get more exposure than I would have maybe through just our regular rotations. (Cohort 4)
Impact on Residency Applications	I think it definitely helped me in terms of applying for residency and being able to speak on my research experience and knowing what I did like and what I didn’t like about research and how I’d like my research career to look. I think I just had more experience than I would have had otherwise to be able to speak on that. (Cohort 4) When I went on some interviews for places that [my mentor] knew the leadership well, I showed up for my interview and they already knew my name, and I think all of those things are very, very helpful and probably would not have happened otherwise. (Cohort 2)
Impact on Interest in Research	I think professionally it was also incredibly helpful in various ways because it got me involved in research early and allowed me to get connected to various people and become involved in various projects. (Cohort 4) We got to work on many different cool projects together that I got publications and presentations from so overall I loved it, and I thought it worked out very well for me at least. (Cohort 4) I think that if I hadn’t done the METEOR program I don’t think I’d be as interested … I know I have an interest in research, but I wouldn’t know how to navigate. I would know what it would look like one, and then I don’t think I would have had the resources to actually go through and do it. (Cohort 1)
General Support beyond Research	As far as academics go, I remember having a hard time getting through [USMLE] Step 1, I think just like any other medical student, but what was very helpful was my mentor sat down and gave me his schedule for Step 1 as well as for Step 2, and he said ‘Look these are the things that worked for me, this didn’t work for me, they may work for you, they may not work for you, but this is how I tackled that situation.’ And he did the same thing with classes. So he said ‘Like this is how I took notes, this is how I process information, this book was helpful, this book wasn’t helpful.’ I think that guidance really helped me. (Cohort 1) I definitely think he was incredibly committed to my development not just as a researcher but as a physician (Cohort 3) I think it definitely got me a lot more comfortable in terms of the faculty there and their investment in our professional development, and it significantly made me comfortable starting medical school. (Cohort 3) Part of resiliency is having a couple people that I felt like I could count on, people to talk to you and even just people that believed in me. So like sometimes you’re going through a lot of things, it’s easy to kind of lose confidence in who you are or your ability to get through it, but when you have people who are seasoned and attendings and such that see you and know you and still believe in you anyway, it’s very helpful. (Cohort 2)

Several participants appreciated seeing their mentor successfully integrate research into their clinical practice:

Working with a physician-scientist, it was neat to see how he balances clinic and research, and how he works with people that are doing basic research, but he himself does more of the clinical side of things. And so I think that’s the kind of research I would like to do in the future. I think that I’m definitely more interested in clinical versus bench [research], and so it was nice to see how he makes it work and how he’s able to divide his time to do both. (Cohort 4).

Participants gained a broader perspective of the overall process of research than they had been previously exposed:

I had come in with a little bit of knowledge of bench work, but [METEOR] definitely exposed me to research in terms of a broader sense, in terms of just not doing bench work and doing more clinical stuff, which I had never been exposed to before … seeing what the process is like and how long it can take to have something published or to study something or the different steps and the different team members involved … great exposure to the nitty-gritty that you might not expect or you might not know that research entails, especially if you haven’t had a lot of experience coming in. (Cohort 4).

Mentors were instrumental in assisting participants in choosing a specialty:

I was not certain what specialty I was going into. [My mentor] definitely contacted people from different specialties and allowed my time there to send me to work with those specialties to help me get exposure, to kind of figure out what my interest was … He very much advocated for being exposed to as many specialties as I could and helped facilitate that whenever he could as well, which was great. (Cohort 3).

The opportunity to join the GW community prior to the start of medical school and to be part of a cohesive cohort were frequently noted as being beneficial:

I’ve developed a relationship with a mentor from the very start, and it set the tone for learning how to establish yourself as a researcher and how to reach out to make connections before I started medical school. (Cohort 1).

Similarly, a member of Cohort 4 noted:

It’s so incredibly helpful that you’re able to move in a little earlier; you meet some people in this new city, but at the same time you get to explore the city. We did so many neat things that first summer just in terms of going to lectures at Children’s but then also doing site visits at the FDA and things like that, so I think it’s great exposure.

Overall mentorship was seen as a key component of the METEOR Program and many relationships continued after graduation into residency:

I overall thought it was great, not just the research but the mentorship. [My mentor] still actually emails me like every month or two, and we still are able to have conversations about just medicine. (Cohort 3)

## Next steps

This report focuses only on the first four cohorts of students who completed the METEOR program. Our findings will be incorporated into improvements to the program for future cohorts, and with increased enrollment, quantitative studies of the effectiveness of the program are planned.
